# Disparities in Adherence to End‐of‐Life Care Directives: The Role of Cognitive Status and Race/Ethnicity

**DOI:** 10.1002/alz70858_103977

**Published:** 2025-12-25

**Authors:** Zahra Rahemi, Juanita‐Dawne Bacsu, Matthew Lee Smith, Elham Hariri Kashani, Swann Arp Adams

**Affiliations:** ^1^ Clemson University, Greenville, SC, USA; ^2^ University of Saskatchewan, Saskatoon, SK, Canada; ^3^ Texas A&M University, College Station, TX, USA; ^4^ Kashan University of Medical Sciences, Kashan, Isfahan, Iran (Islamic Republic of); ^5^ University of South Carolina, Columbia, SC, USA

## Abstract

**Background:**

Alzheimer's disease and related dementias (ADRD) are a leading cause of death and the most costly disease in the U.S., with end‐of‐life costs surging dramatically in the final months. Advance care planning (ACP) is critical to align care with patient goals, yet completion rates remain low, especially among marginalized groups like Black and Hispanic patients, who face systemic disparities in care. While ACP completion upstream is essential, it is unclear if care at end of life aligns with patients’ documented preferences, particularly for those with cognitive impairment. This study addresses these critical gaps by exploring disparities in ACP adherence at end of life.

**Method:**

Data for this study were sourced from the Health and Retirement Study (HRS), including the RAND Harmonized Exit File, Harmonized HRS‐D, and HRS Tracker File (1995–2016). The Exit File consists of interviews conducted with proxy respondents for individuals who completed at least one “alive” interview and subsequently died. Cognitive status was assessed based on a proxy‐reported rating of the decedent's memory approximately one month prior to death, categorized as either “poor” or “normal” cognition. Adherence to end‐of‐life directives was evaluated using a question that inquired whether any issues arose in following the decedents’ written instructions.

**Result:**

The majority of participants in the cohort were urban, White, non‐Hispanic, had a high school education or less, and not married. Twenty‐six percent of the population had a poor memory rating approximately one month prior to their death. We observed significant effect modification by race of the association between cognition and adherence to end‐of‐life directives (*p* = 0.02). Among black participants, the odds of not adhering to end‐of‐life instructions among those respondents with poor cognition were 2.2 times significantly higher than those who had normal cognition (95% CI: 1.02, 4.75). No significant associations were demonstrated in the white or other racial groups.

**Conclusion:**

Racial disparities in adherence to end‐of‐life directives may be influenced by cognitive status, with potential implications for care alignment in diverse populations. This study highlights the need to address both cognitive decline and racial disparities to improve the implementation of patient preferences and enhance end‐of‐life care outcomes.

